# How the COVID-19 Pandemic Affects the Provision of Psychotherapy: Results from Three Online Surveys on Austrian Psychotherapists

**DOI:** 10.3390/ijerph20031961

**Published:** 2023-01-20

**Authors:** Stefanie Winter, Andrea Jesser, Thomas Probst, Yvonne Schaffler, Ida-Maria Kisler, Barbara Haid, Christoph Pieh, Elke Humer

**Affiliations:** 1 Department for Psychosomatic Medicine and Psychotherapy, University for Continuing Education Krems, 3500 Krems, Austria; 2 ABILE-Viktor Frankl Education Austria, 3390 Melk, Austria; 3 Austrian Federal Association for Psychotherapy, 1030 Vienna, Austria

**Keywords:** COVID-19, psychotherapy, teletherapy, remote psychotherapy, patient numbers

## Abstract

This study aimed to assess patient numbers and the format in which psychotherapy was delivered by Austrian psychotherapists during different time points of the COVID-19 pandemic and to explore psychotherapists` experiences on pandemic-associated changes in their psychotherapeutic work as well as their wishes for support in their professional activities. Three cross-sectional online surveys were conducted between March 2020 and May 2022. The total number of participating psychotherapists was *n* = 1547 in 2020, *n* = 238 in 2021, and *n* = 510 in 2022. The number of patients treated was highest in 2022 and lowest at the beginning of the pandemic (*p* < 0.001). During the lockdown in 2020, only 25.0% of patients were treated in personal contact. This proportion increased in the following years, reaching 86.9% in 2022 (*p* < 0.001). After a substantial increase in the proportion of patients treated via the telephone and internet during the first lockdown, both proportions decreased during the pandemics’ second and third year (*p* < 0.001). However, a larger proportion of patients were treated via the internet in 2022 compared to pre-pandemic times (*p* < 0.001). Psychotherapists reported that the pandemic affected mainly the setting in which psychotherapy was provided (29.6%), the working conditions and workload (27.1%), as well as the demand for psychotherapy (26.9%). About one-third of psychotherapists expressed support wishes for their psychotherapeutic work. Results suggest that the pandemic went along with a partial shift in the provision of psychotherapy towards psychotherapy via the internet but not the telephone. The increase in patient numbers and psychotherapists` reports of increased workload suggest a rise in the demand for mental health care during and in the aftermath of the pandemic.

## 1. Introduction

With the outbreak of the Coronavirus disease 2019 (COVID-19), many mental healthcare providers discontinued face-to-face psychotherapy to reduce the risk of infection [1,2,3]. At the same time, the prevalence of mental health disorders (i.e., depression, anxiety, insomnia) increased in the general population [4,5,6]. Thus, it is assumed that the demand for psychotherapy increased compared to pre-pandemic times [1]. To enable the treatment of their patients, many psychotherapists replaced some of their face-to-face contacts with therapies conducted at a safe distance, i.e., via telephonic communication and videoconferencing [7]. Despite two decades of evidence-based remote psychotherapy demonstrating robust clinical effectiveness being not different from in-person settings [8], several barriers prevented its implementation in clinical practice before the pandemic [9,10]. The COVID-19 pandemic has been expected to cause a wide-scale acceptance of remote psychotherapy and to lead to a part-robust shift in the format psychotherapy is provided towards treatment via the internet [1]. A previous study conducted in Austrian psychotherapists revealed that after one year of the pandemic, most patients were treated again in the traditional face-to-face setting [11]. Nevertheless, more patients were treated via remote formats than before the pandemic. Whether the proportion of patients treated from a distance decreased to pre-pandemic levels with the ongoing pandemic has not been assessed so far. Thus, the first aim of this study was to determine the number of patients treated as well as the format in which Austrian psychotherapists delivered psychotherapy during different time points of the COVID-19 pandemic (from the first months to the third year of the pandemic) and the potential differences compared to pre-pandemic times. We were also interested in whether differences exist concerning the gender and therapeutic orientation of the psychotherapists, as previous studies have shown differences in the treatment format used between male and female psychotherapists [12] and in the experiences with remote settings among therapists with different psychotherapeutic orientations [13].

Additionally, we aimed to put this information on the changes in patient numbers and the therapeutic format in context by exploring psychotherapists’ experiences of the effect of the COVID-19 pandemic on their psychotherapeutic work using content analysis.

During the first weeks of the pandemic, a study evaluating information needs regarding internet use in psychotherapy revealed that several psychotherapists wished for further information on data protection and security issues [14]. To explore possible wishes of psychotherapists for further support in a broader context, a third aim was to evaluate support wishes regarding their professional activities in general. To provide adequate mental health care in the future, it is vital to have the voices of psychotherapists be heard as well. Their statements can help to gain a better insight into issues of their professional work and to identify flaws, and the possibilities of reviewing and improving the current psychotherapeutic system in Austria. This study aimed to fill existing research gaps, and it is also hoped that it will help professional associations to use its findings to improve the representation of their members’ interests and to give them the chance to participate in future decisions.

## 2. Materials and Methods

Between March 2020 and May 2022, 3 cross-sectional online surveys on Austrian psychotherapists were conducted using Research Electronic Data Capture (REDCap) (Vanderbilt University, Nashville, TN, USA) [15]. The common aim of the surveys was to assess the number of patients treated per treatment format during different times of the pandemic. Next to this common aim, each of the surveys aimed at different topics, which will shortly be described in the following, but are not part of the current study.

The first survey was open between 24 March and 1 April 2020, the time of the first wave of COVID-19 infections in Austria, which was accompanied by a strict nationwide COVID-19 lockdown [16]. The main aim of the survey was to assess the lockdown-related changes in the provision of psychotherapy and experiences of psychotherapists with remote settings. The survey comprised 79 items. Detailed information on the conduction of the study and the obtained results on the provision of psychotherapy, experiences of psychotherapists with remote psychotherapy, as well as perceived stress levels and job anxieties of psychotherapists, have been published previously [7,13,14,17].

The second survey was open from 16 February to 2 April 2021 and comprised 40 items. The main aim of the survey was to assess the provision of psychotherapy during the second year of the pandemic as well as on attitudes toward evidence-based practice and self-assessment bias. During the survey, the third wave of infection (the Alpha variant) hit Austria. Daily confirmed COVID-19 cases were high, and several regionally adapted lockdown measures were in place [18]. We refer to the already published work for more detailed information on the study design and obtained results [11,19,20].

The third survey was conducted between 11 April and 31 May 2022. During this time, the Omicron variant was dominant in Austria, which led to new highs in daily confirmed COVID-19 cases. Due to the milder course of the Omicron variant, these high infection rates did not lead to direct congestion of healthcare facilities, and measures to contain the spread of the virus were strongly relaxed at the time of the third survey [21]. The survey constituted 50 items. The main aim of the survey was to assess the provision of psychotherapy during the third year of the pandemic as well as on mental health in psychotherapists. More information on the recruitment of the participating psychotherapists has been published before [22].

The studies were conducted following the Declaration of Helsinki and approved by the Ethics Committee of the University for Continuing Education Krems, Austria (Ethical numbers: EK GZ 2018–2021, EK GZ 11/2021–2024). All participants gave electronic informed consent to participate and complete the questionnaires. Psychotherapists` participation was voluntary, without incentives. To ensure strictly anonymous data collection, no personal data to identify psychotherapists were collected. Thus, merging data from the 3 surveys and considering a potential participant overlap was impossible.

### 2.1. Measures

#### 2.1.1. Sociodemographic Variables

All participants were asked about their gender, age, and years in the profession (defined as the time since they were registered in the official list of licensed psychotherapists). They were also asked about the psychotherapeutic method in which they were trained. The surveys conducted in 2020 and 2022 inquired using a list of all 23 methods accredited in Austria [23]. In the survey conducted in 2021, the broader category of the 4 orientations (psychodynamic, humanistic, systemic, behavioral) was asked for. For further analyses, data on the 23 specific methods gathered in 2020 and 2022 were classified into 4 orientations. In 2021, a small number of psychotherapists in training under supervision participated. Their professional years were classified as “0” for further analysis.

#### 2.1.2. Number of Patients Treated and Psychotherapeutic Format

In all 3 surveys, participating psychotherapists were asked about the number of patients treated on average per week. In the first survey, this question was asked for 2 different time frames: the months before the COVID-19 lockdown (retrospectively) as well as since the COVID-19 lockdown. In 2021 and 2022, the question was only related to the time of the study. Psychotherapists were asked to provide the number of patients treated on average per week for 3 different treatment formats separately: in personal contact, via the internet, and the telephone. Data on the number of patients treated per treatment format collected in 2020 and 2021 have been published previously [7,11] and were re-analyzed together with the data gathered in 2022 in the study at hand to investigate changes in the number of patients as well as treatment format over a broader time frame.

For further analyses, data were summarized per time point (2020 before lockdown, 2020 during lockdown, 2021, 2022) and psychotherapist to receive the total number of patients treated per psychotherapist per week and time point. The numbers per treatment format and time point per psychotherapist were related to the total number of patients treated per time point and psychotherapist to adjust the data for the different patient numbers over time.

#### 2.1.3. Effects of the Pandemic on Psychotherapeutic Work and Wishes for Support with Professional Activities

To gain a deeper understanding of the psychotherapists` views on the effects of the pandemic on their psychotherapeutic work, the latest survey (conducted in spring 2022) included the following free text question: “What direct or indirect effects did the pandemic have on your work as a psychotherapist?”.

A further question asked: “Would you wish support concerning your professional activity as a psychotherapist?”. All participants who answered this question in the affirmative were asked open-endedly to explain in a text box what form of support would be helpful for them (“What support concerning your professional activity as a psychotherapist would you wish for?”).

### 2.2. Statistical Analyses

Descriptive statistics were conducted to describe sociodemographic characteristics. Chi-squared tests and univariate analysis of variance tests (ANOVAs) were applied to assess differences in sociodemographic and professional characteristics between participating psychotherapists in the different years (2020, 2021, 2022).

As only 1 gender-diverse psychotherapist participated in the 3 surveys, statistical analyses were conducted only on male and female psychotherapists. Furthermore, psychotherapists who could not be assigned to 1 of the 4 therapeutic orientations were excluded from further analyses.

Univariate ANOVAs were computed to assess differences in the total number of patients treated per psychotherapist and week, as well as differences in the proportion of patients treated per psychotherapist in personal contact, via the internet, or by telephone. The initial model included time (2020 before lockdown, 2020 during lockdown, 2021, 2022), gender (female, male), therapeutic orientation (psychodynamic, humanistic, systemic, behavioral), and their interactions as independent variables. Age and professional experiences were included as covariates in the statistical model. As all investigated dependent variables did not differ among the 4 therapeutic orientations, this variable was removed from the final model.

Chi-squared tests were conducted to analyze potential differences in the proportion of psychotherapists wishing further support with their professional activities between male and female psychotherapists and between the 4 therapeutic orientations.

Statistical analyses were performed in SPSS version 26 (IBM Corp, Armonk, NY, USA). *p*-values of <0.05 were considered statistically significant (2-sided tests).

### 2.3. Content Analyses

The free-text answers were evaluated using content analysis [24] with subsequent quantification of qualitative categories and coded with the Atlas.ti software [25]. A total of 626 free text comments were received; 466 related to the experienced changes in psychotherapeutic work and 160 referred to the wishes of psychotherapists regarding their professional activities. The length of the replies differed from one word up to several sentences. As an example, on the question about the experienced changes, some psychotherapists (*n* = 30) replied simply with “none”. The comments were read multiple times by one researcher to get an overview of the data. In the next step, categories and coding rules were defined and discussed in the research team. Then one researcher started coding the whole dataset. Each code was applied between 3 and 151 times, the total of applied codings was 1965. In this process, 14 main categories and 30 subcategories gradually emerged inductively. In the next step, the strength of the categories was defined depending on the frequency of participants who endorsed them so that the magnitude of the individual themes appears more clearly. While the main categories are visualized graphically in the results section, the subcategories are mentioned exclusively in the accompanying text. As some of the more detailed text passages related to different aspects of the open-ended questions, they were assigned to several codes. An example of an open-ended question that was assigned to several codes is the answer of case 234 on the question regarding the experienced changes: “More frequent postponements of appointments due to infected clients, increased demand, change in the relationship (no handshaking, different attitudes of the persons regarding the handling of the pandemic), increased caution in dealing with clients (inner tension, fears)”. To ensure an intersubjective understanding of categories and sharpen the coding rules, intermediate findings were discussed with another researcher several times.

## 3. Results

### 3.1. Study Sample Characteristics

The total number of participating psychotherapists was highest in 2020 (*n* = 1547) and lowest in 2021 (*n* = 238; Table 1). Mean age and professional experience were highest in psychotherapists participating in 2022 (*p* ≤ 0.028). Among all surveys, humanistic psychotherapists were the largest group. Differences among years (*p* = 0.046) revealed that their relative proportion was highest in 2021, making up for more than half of the psychotherapists.

### 3.2. Changes in the Total Number of Patients

**Figure 1:**
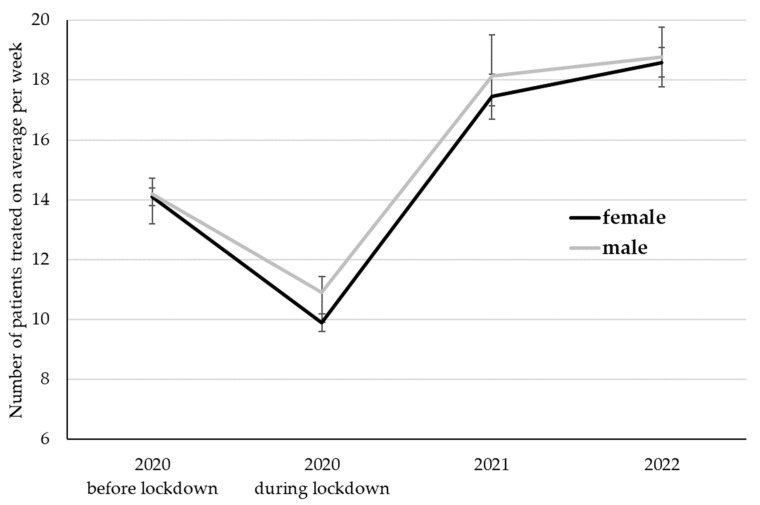
The number of patients treated on average per week. LS Mean ± standard error.

The total number of patients treated differed among time points (*p* < 0.001) but was neither affected by gender (*p* = 0.35) nor the interactions between time and gender (*p* = 0.74; Figure 1). Bonferroni-corrected post hoc tests revealed higher numbers of treated patients in 2022 and 2021 compared to 2020 before as well as during the lockdown (*p* < 0.001). The lowest numbers were observed during the first COVID-19 lockdown in 2020, differing from all other time points (*p* < 0.001).

### 3.3. Changes in Treatment Format

**Figure 2:**
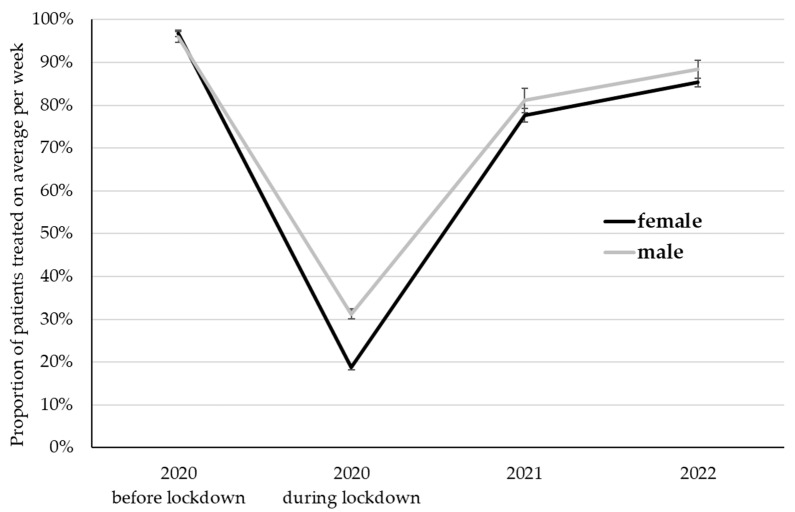
The proportion of patients treated on average per week in personal contact. LS Mean ± standard error.

The proportion of patients treated in personal contact differed among time points (*p* < 0.001), female and male psychotherapists (*p* < 0.001), and was affected by the interaction of time and gender (*p* < 0.001; Figure 2). Bonferroni-corrected post hoc tests revealed significant differences among all time points (*p* ≤ 0.001), with the highest proportions before the lockdown in 2020 (96.3%). During the lockdown in 2020, only 25.0% of patients were treated in personal contact, and this proportion increased in the following years to 79.3% in 2021 and 86.9% in 2022. Among all time points, female psychotherapists treated 69.6% of their patients in personal contact, which is lower compared to the 74.1% of male psychotherapists. A closer look at the differences among time points reveals that significant differences among female and male psychotherapists were only pronounced during the first lockdown in 2020 (18.8% in females vs. 31.2% in male psychotherapists; *p* < 0.001).

**Figure 3:**
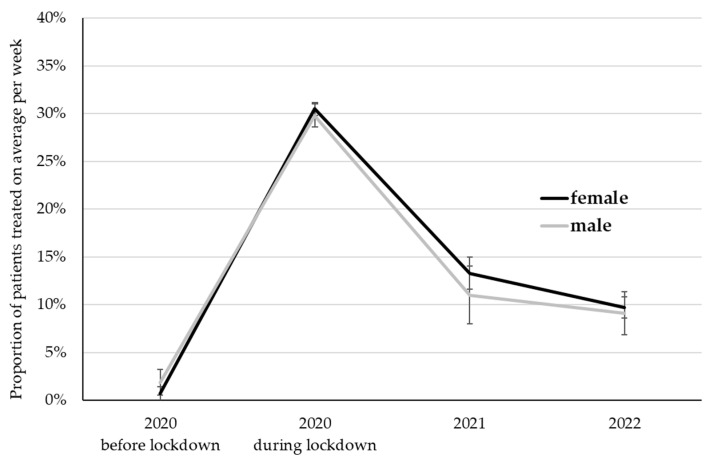
The proportion of patients treated on average per week via the internet. LS Mean ± standard error.

The proportion of patients treated via the internet differed among time points (*p* < 0.001) but was not affected by gender (*p* = 0.61), as well as the interaction between time points and gender (*p* = 0.72; Figure 3). The highest proportion was observed during the lockdown in 2020 (30.1%), differing from all other time points (*p* < 0.001). This proportion decreased to 12.2% (2021) and 9.4% (2022) in the following years. Despite the substantial decline with the prolongation of the pandemic, the proportion of patients treated via the internet in 2022 was more than seven-fold as high as before the pandemic (1.3%; *p* < 0.001).

**Figure 4:**
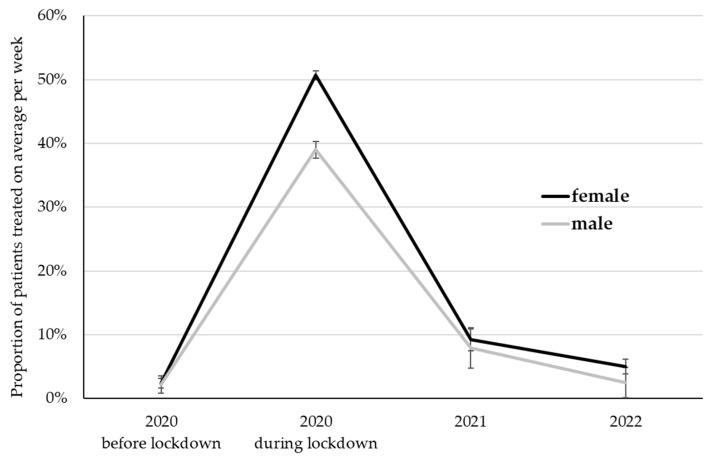
The proportion of patients treated on average per week via the telephone. LS Mean ± standard error.

The proportion of patients treated via the telephone differed among time points (*p* < 0.001), female and male psychotherapists (*p* = 0.002), and was affected by the interaction of time and gender (*p* < 0.001; Figure 4). Bonferroni-corrected post hoc tests revealed significantly higher proportions of patients treated via the telephone during the first lockdown (44.9%) compared to all other time points (*p* < 0.001). This proportion declined to 8.5% in 2021 and 3.8% in 2022. While the proportion in 2021 exceeded pre-pandemic values (*p* = 0.01), it reached similar values in 2022 compared to the time before the first lockdown (2.3%; *p* = 1.00). Averaged among time points, female psychotherapists treated a higher proportion of their patients via the telephone (16.8%) than their male colleagues (12.9%; *p* = 0.002). Bonferroni-corrected pair-wise comparisons between male and female therapists within time points revealed that these gender differences were only pronounced during the first lockdown in 2020 (50.7% in female vs. 39.0% in male psychotherapists; *p* < 0.001).

### 3.4. Free-Text Answers on Experienced Changes in Psychotherapeutic Work

**Figure 5:**
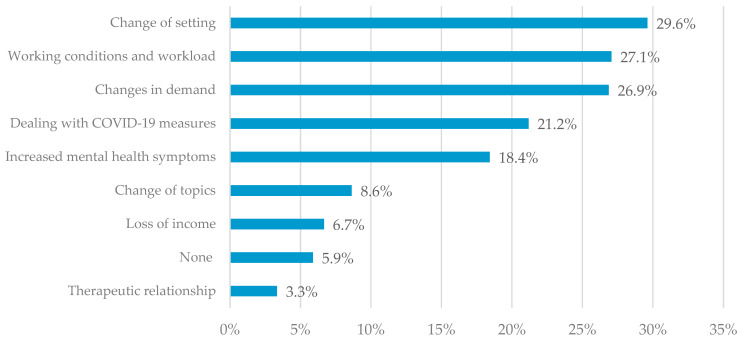
Experienced changes in psychotherapeutic work. The percentages of psychotherapists reporting each main category of response that emerged from the open-ended question, “What direct or indirect effects did the pandemic have on your work as a psychotherapist?”

A total of *n* = 466 (91.4%) psychotherapists participating in the survey conducted in 2022 gave a valid answer to the open-ended question on the direct or indirect effects of the pandemic on their psychotherapeutic work. Content analyses resulted in 9 main categories (Figure 5) and 19 subcategories.

The most frequent comments concern the category “change of setting” (*n* = 151; 29.6%), referring to changes in treatment format, mainly due to an increase in remote therapy (*n* = 145). Most therapists (*n* = 86) commented neutrally about remote psychotherapy, *n* = 47 positively, and *n* = 12 negatively. Another 6 of the 151 psychotherapists who described changes in the setting reported treating their patients outdoors as an alternative to holding a session in their practice.

*“Patients, after periods of doubt, enjoyed online psychotherapy very much, and the effect is 100% equal to that in face-to-face”*.

(Case 329)

*“I found the therapeutic work via Zoom much more exhausting and less effective than face-to-face contact. Some interventions were not possible via Zoom”*.

(Case 410)

Furthermore, 138 (27.1%) psychotherapists reported that the pandemic affected their working conditions and workload. While *n* = 129 expressed an increased workload, *n* = 9 reported a decrease. The main topics were time flexibility, dealing with last-minute cancellations and COVID-19 measures, fear of COVID-19 infection, facing the same challenges as patients, and less time for recovery.

A further 137 (*n* = 26.9%) responses related to changes in demand, with the majority (*n* = 104) reporting an increase and n = 33 reporting a decrease. Psychotherapists reported increased requests from new and former patients whose therapeutic process had already been completed. As some therapists could not cope with the number of requests, they had to deal with waiting lists or reject patient requests.

Dealing with COVID-19 measures in the practice (i.e., face mask mandates) also appeared as a topic (*n* = 108; 21.2%). A total of *n* = 57 respondents signaled compliance in this regard, *n* = 42 reported challenges to adhering to the COVID-19 measures, 6 felt uncertain about them, and 3 stated that they did not comply with the COVID-19 measures.

*“My facial expressions are missed by the clients; eating disorders are recognized much later, hiding behind the mask”.*

(Case 38)

An increase in mental health symptoms was mentioned by *n* = 94 (18.4%). Psychotherapists stated that they perceived patients to be more stressed and that more and more children, adolescents, and young adults were seeking therapy. They also reported increased anxiety disorders, depression, eating disorders, and suicidal thoughts.

*“I have noticed a massive deterioration in depressed and eating disordered clients and increased stress in very young people who were previously completely free of symptoms”.*

(Case 153)

Furthermore, *n* = 44 (8.6%) reported a change of topics. Psychotherapists stated that the focus of the therapy moved to current events, such as dealing with the pandemic and the Russia–Ukraine conflict (*n* = 24). In addition, *n* = 20 psychotherapists reported that COVID-19 became a conflict topic in the therapeutic setting.

*“The simultaneousness of the shared experience, but with very different attitudes towards them, sometimes caused anger against patients, e.g., when they worried about compulsory tests for skiing or about measures and vaccinations in general. To always maintain professional restraint in such cases was almost equivalent to self-harm for me over time“*.

(Case 475)

Another *n* = 34 (6.7%) psychotherapists described a loss of income due to infection with COVID-19 (*n* = 22) or due to reduced demand (*n* = 12).

*“At the beginning, I had big financial losses. Currently, it has decreased, but I have at least 3–5 cancellations per week”*.

(Case 136)

A small proportion (*n* = 30; 5.9%) of the participating psychotherapists stated that they did not notice any changes in their psychotherapeutic work.

Effects on the therapeutic relationship also emerged (*n* = 17; 3.3%), with the majority (*n* = 12) reporting adverse effects on the therapeutic relationship, while the remaining *n* = 5 sensed an improvement.

*“Due to wearing a mask, loss of mimic for expressing emotions. On the other hand, stress reduction for clients with personality disorders… dissociative symptoms”*.

(Case 256)

*“More bonding with the clients “we are all in the same boat”.*

(Case 149)

### 3.5. Free-Text Answers on Wishes for Support in Psychotherapeutic Work

In the latest survey, participants were also asked for support wishes concerning their professional activities. A total of *n* = 175 (35.1%) answered “yes” to this question, with no differences between male and female therapists (χ^2^ (1) = 0.54; *p* = 0.46). No differences among the therapeutic orientations emerged (χ^2^ (3) = 0.765; *p* = 0.86).

**Figure 6:**
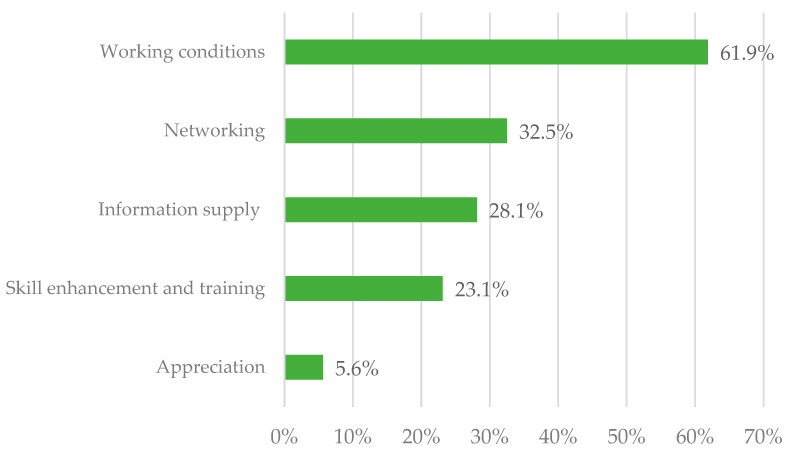
Support wishes. The percentages of psychotherapists reporting each main category of response that emerged from the open-ended question, “What support concerning your professional activity as a psychotherapist would you wish for?”

The majority (*n* = 160; 91.4%) of psychotherapists who stated that they wish for further support gave a valid answer to the open-ended question on which kind of support they would like to receive. Content analyses resulted in 5 main categories (Figure 6) and 11 subcategories.

The main category “working conditions” was endorsed by 61.9% (*n* = 99) of the psychotherapists providing a valid answer to this question. Psychotherapists referred to improved funding (*n* = 62), less bureaucracy (*n* = 19), financial security in the event of loss of earnings (*n* = 8), administrative support (*n* = 7), and technical support (*n* = 3).

*“I feel that, for financial reasons, patients only seek psychotherapy when the subjective sense of a disorder is already very severe. Many clients for whom psychotherapy would be essential cannot yet afford it“.*

(Case 353)

Another *n* = 52 (32.5%) psychotherapists expressed their wish for more networking among their discipline (*n* = 42) and multidisciplinary networking (*n* = 10).

*“Exchange forums for dealing with the current situation“.*

(Case 179)

*“Keynotes or something similar on current topics (cross-school networking) (e.g., dealing with the pandemic), easy and low-threshold access possible especially via the use of video telephony”.*

(Case 234)

*“More exchange and cohesion instead of competition”.*

(Case 344)

Information provision was stated by 28.1% (*n* = 45). Of these, *n* = 23 mentions related to more support from the professional associations (i.e., the Austrian Federal Association for Psychotherapy (ÖBVP) and the Association of Austrian Psychotherapists (VÖPP)).

*“A contact to which one can turn, for example, regarding newly prescribed measures. ÖBVP and VÖPP were and are only of limited help”.*

(Case 102)

Psychotherapists also mentioned a need for information regarding the handling of COVID-19 restrictions (*n* = 8) and COVID-19 conspiracists and critics (*n* =5). Psychotherapists also stated that it would be important to be more aware of options for mental hygiene and psychoprophylaxis (*n* = 9).

Furthermore, 37 (23.1%) answers were related to skill enhancement and training. This main category refers to psychotherapists’ wishes for more free-of-charge or affordable group and individual supervision, advanced training, and meetings on current topics and regulations.

Lastly, a few psychotherapists (*n* = 9; 5.6%) mentioned that they wished for more appreciation of their work.

## 4. Discussion

A major finding was that after a decline in the total number of patients treated during the first weeks of the pandemic, patient numbers increased and exceeded pre-pandemic levels during the second and third year of the pandemic. In accordance, results from the free-text question on the effect of the pandemic on psychotherapeutic work suggest that after an initial decline in patient numbers during the first weeks of the pandemic, a large proportion of psychotherapists experienced an increased demand for psychotherapeutic services, a higher workload, as well as an increase in mental health symptoms in their patients. These findings suggest a continuous rise in the need for psychotherapeutic services during the pandemic, corroborating the alarming results observed regarding the mental health status of the Austrian general population during the past 2.5 years. During the first weeks of the pandemic (April 2020; during the first COVID-19 lockdown), a substantial increase in the prevalence of mental health symptoms was observed in the Austrian general population (21% depression, 19% anxiety, 16% insomnia) [6]. Mental health symptoms further increased at the end of the first year of the pandemic (December 2020/January 2021), during the third COVID-19 lockdown (26% depression, 23% anxiety, and 18% insomnia [26]). During the third year of the pandemic—in spring 2022—COVID-19 restrictions were strongly lifted. Nevertheless, mental health symptoms remained at a high level in April 2022, showing even a further increase in the prevalence of depressive symptoms (28%) and no significant change for anxiety (16%) and insomnia (15%) compared with April 2020 [27].

Another important finding was that, after a substantial increase in the proportion of patients treated via the telephone and internet during the first lockdown, both proportions decreased during the second and third years of the pandemic. The proportion of patients treated via telephone declined to pre-pandemic levels during the third year of the pandemic, while the proportion of patients treated via the internet in 2022 was seven times higher than in 2020. Therefore, it seems that psychotherapists learned to appreciate the advantages of psychotherapy provided via the internet, such as local flexibility, reduced travel time and costs, and providing access to care in underserved locations [28], resulting in a significant proportion of patients being treated in this format even at the begin of the third year of the pandemic. Therefore, it seems likely that the increase in online psychotherapy as a response to this public health emergency will be a stable change. This observation is supported by the answers to the free-text question regarding experienced changes due to the pandemic. Almost one-third of the participating therapists reported changes in the setting, i.e., remote therapy, with the majority reporting this change as neutral (59%) or positive (32%). However, some psychotherapists reported impairment in the therapeutic relationship and missing direct contact. Although previous studies point to a similar efficacy of remote psychotherapy vs. in-person psychotherapy [8,29,30], the suitability of remote psychotherapy might differ among psychiatric disorders. While remote settings have been suggested to be less appropriate in acute crises or for people with severe psychiatric disorders [28,31], patients with avoidant personality traits or those dealing with body-image disorders or sexual abuse might benefit from remote settings [32,33,34]. As research on the suitability of remote settings for different psychiatric disorders is inconclusive, further research is required to be able to make clear recommendations. To ensure adequate engagement in psychotherapy via the internet, psychotherapists need to receive adequate training in delivering interventions online and adequate knowledge on potential contraindications and on legal aspects (i.e., data protection issues), as well as a certain minimum level of digital literacy and technological competence [14,28].

Gender differences in the proportion of patients treated in personal contact as well as via the telephone became evident, showing a higher proportion of female psychotherapists treating their patients via the telephone at the expense of in-person psychotherapies during the first lockdown in 2020. The lower proportion of patients treated in personal contact by female psychotherapists during the first COVID-19 lockdown might be explained by a higher fear of COVID-19 infection in psychotherapies provided face-to-face in female vs. male psychotherapists, as reported previously in Czech, German, and Slovak psychotherapists [12]. Another reason might be the higher willingness of women to adhere to protective measures [35,36]

Opposite to gender, all investigated variables did not differ concerning therapeutic orientation. This result is in line with previous studies, revealing no relevant differences in the therapy format provided during the COVID-19 pandemic between psychodynamic, humanistic, behavioral, and systemic psychotherapists [7,11].

Given the increased demand placed on psychotherapists, we also aimed to investigate possible wishes for support with professional activities, which psychotherapists suggested via answers to open-ended questions. To enable adequate mental healthcare in the future, higher reimbursement of treatment costs by insurance, less bureaucracy, more networking among psychotherapists, and more information from professional associations of psychotherapists have been suggested. Other Austrian studies have addressed these aspects as well [37,38]. Comments indicate that the demand for more affordable psychotherapy is increasing and that, in some cases, urgently needed psychotherapy is discontinued or not even started due to financial reasons. Next to financial constraints, psychotherapists reported that some patients struggle with the complex bureaucratic procedures associated with applications for reimbursement of treatment costs.

Around one-third of the therapists who stated desired support with their professional activities expressed their wish for more professional exchange among their discipline and in multidisciplinary networking. To encourage and facilitate such interaction between professionals, intervision-groups with participants from various therapeutic orientations or networking events should be considered to address this need. It is unclear to what extent competitive pressure among self-employed psychotherapists may have influenced their previous interaction and whether the wish for more networking only became noticeable because of the pandemic and the accompanying changes in practice. In general, responses indicate excellent potential for self-employed psychotherapists to perceive themselves as a source of support rather than as competitors.

Recurring topics were handling the COVID-19 guidelines and their implementation in practice, as well as coping with new regulations. To minimize uncertainty and resentment about decreed regulations, therapists suggested more rapid and user-friendly information on the websites of professional associations. Provision of self-printable information material, e.g., on COVID-19 regulations for private practices, and more frequent offers of affordable or free online seminars on changes in current regulations have been suggested.

This study has potential limitations. First, the strictly anonymous data collection did not allow merging the data from the 3 cross-sectional surveys on an individual level. Thus, no information regarding repeated measures is available, i.e., the psychotherapists participating in 2020, 2021, and 2022 could be completely different among time points, or there could be some overlap. Second, data from the weeks before the first COVID-19 lockdown were collected retrospectively and might be subject to recall biases. Third, the online data collection might have caused a participation bias toward psychotherapists with a higher affinity toward digital media. Fourth, the number of participants differed strongly among surveys. Although recruitment did not differ among surveys it can be assumed that the different main topics of the surveys have led to different response rates. While the topic of experiences with remote settings and the provision of remote psychotherapy was a relevant topic for almost all psychotherapists during the first survey due to the first nationwide strict lockdown rules in place during that time, the theme of the second survey (attitudes toward evidence-based practice and self-assessment bias) was likely of lower personal interest to the psychotherapists.

## 5. Conclusions

After an initial decline in face-to-face psychotherapy during the first COVID-19 lockdown, most patients were treated in the conventional face-to-face setting during the second and third years of the pandemic. Nevertheless, results suggest that the COVID-19 pandemic went along with a partial shift in the provision of psychotherapy towards psychotherapy via the internet. The increase in patient numbers and psychotherapists` reports on increased workload further suggests a rise in the demand for mental health care. To enable adequate mental health care in the future, higher reimbursement of treatment costs by insurance, less bureaucracy, more networking among psychotherapists, and more information from professional associations of psychotherapists have been suggested.

## Figures and Tables

**Table 1:** Study sample characteristics.

Variable	2020	2021	2022	Statistics
	(*n* = 1547)	(*n* = 238)	(*n* = 510)	
Gender				
Female, % (*n*)	75.7% (1171)	76.9% (183)	80.6% (411)	χ^2^ (4) = 13.84
Male, % (*n*)	24.3% (376)	22.7% (54)	19.4% (99)	*p* = 0.008
Diverse, % (*n*)	0	0.4% (1)	0	
Age in years, M (SD)	51.67 (9.69)	50.97 (9.88)	53.03 (9.94)	F(2;95.37) = 4.87; *p* = 0.008
Years in the profession, M (SD) *	11.19 (9.20)	12.10 (9.70)	12.40 (9.90)	F(2;88.60) = 3.59; *p* = 0.028
Orientation				
Psychodynamic, % (*n*)	20.9% (324)	16.4% (39)	20.8% (106)	χ^2^ (8) = 15.74;
Humanistic, % (*n*)	46.3% (716)	50.8% (121)	46.9% (239)	*p* = 0.046
Systemic, % (*n*)	22.0% (340)	20.2% (48)	22.7% (116)	
Behavioral, % (*n*)	9.8% (151)	8.8% (21)	8.2% (42)	
Others, % (*n*)	1.0% (16)	3.8% (9)	1.4% (7)	

* Information was provided by *n* = 1519 psychotherapists in 2020, *n* = 238 psychotherapists in 2021, and *n* = 502 psychotherapists in 2022. The value was set to “0” for all psychotherapists who were not registered in the list of licensed psychotherapists but working in training under supervision (*n* = 17 in 2021).

## Data Availability

The raw data supporting the conclusions of this article will be made available by the authors without undue reservation.

## References

[1] Wind T.R., Rijkeboer M., Andersson G., Riper H. (2020). The COVID-19 Pandemic: The ‘Black Swan’ for Mental Health Care and a Turning Point for e-Health. Internet Interv..

[2] Humer E., Probst T. (2020). Provision of Remote Psychotherapy during the COVID-19 Pandemic. Digit. Psychol..

[3] Witteveen A.B., Young S., Cuijpers P., Ayuso-Mateos J.L., Barbui C., Bertolini F., Cabello M., Cadorin C., Downes N., Franzoi D. (2022). Remote Mental Health Care Interventions during the COVID-19 Pandemic: An Umbrella Review. Behav. Res. Ther..

[4] Salari N., Hosseinian-Far A., Jalali R., Vaisi-Raygani A., Rasoulpoor S., Mohammadi M., Rasoulpoor S., Khaledi-Paveh B. (2020). Prevalence of Stress, Anxiety, Depression among the General Population during the COVID-19 Pandemic: A Systematic Review and Meta-Analysis. Glob. Health.

[5] Mahmud S., Mohsin M., Dewan M.N., Muyeed A. (2022). The Global Prevalence of Depression, Anxiety, Stress, and Insomnia Among General Population During COVID-19 Pandemic: A Systematic Review and Meta-Analysis. Trends Psychol..

[6] Pieh C., Budimir S., Probst T. (2020). The Effect of Age, Gender, Income, Work, and Physical Activity on Mental Health during Coronavirus Disease (COVID-19) Lockdown in Austria. J. Psychosom. Res..

[7] Probst T., Stippl P., Pieh C. (2020). Changes in Provision of Psychotherapy in the Early Weeks of the COVID-19 Lockdown in Austria. Int. J. Environ. Res. Public Health.

[8] Connolly S.L., Miller C.J., Lindsay J.A., Bauer M.S. (2020). A Systematic Review of Providers’ Attitudes toward Telemental Health via Videoconferencing. Clin. Psychol. Sci. Pract..

[9] Vis C., Mol M., Kleiboer A., Bührmann L., Finch T., Smit J., Riper H. (2018). Improving Implementation of EMental Health for Mood Disorders in Routine Practice: Systematic Review of Barriers and Facilitating Factors. JMIR Ment. Health.

[10] Tuerk P.W., Keller S.M., Acierno R. (2018). Treatment for Anxiety and Depression via Clinical Videoconferencing: Evidence Base and Barriers to Expanded Access in Practice. Focus.

[11] Humer E., Haid B., Schimböck W., Reisinger A., Gasser M., Eichberger-Heckmann H., Stippl P., Pieh C., Probst T. (2021). Provision of Psychotherapy One Year after the Beginning of the COVID-19 Pandemic in Austria. Int. J. Environ. Res. Public Health.

[12] Humer E., Pieh C., Kuska M., Barke A., Doering B.K., Gossmann K., Trnka R., Meier Z., Kascakova N., Tavel P. (2020). Provision of Psychotherapy during the COVID-19 Pandemic among Czech, German and Slovak Psychotherapists. Int. J. Environ. Res. Public Health.

[13] Humer E., Stippl P., Pieh C., Pryss R., Probst T. (2020). Experiences of Psychotherapists With Remote Psychotherapy During the COVID-19 Pandemic: Cross-Sectional Web-Based Survey Study. J. Med. Internet Res..

[14] Humer E., Stippl P., Pieh C., Schimböck W., Probst T. (2020). Psychotherapy via the Internet: What Programs Do Psychotherapists Use, How Well-Informed Do They Feel, and What Are Their Wishes for Continuous Education?. Int. J. Environ. Res. Public Health.

[15] Harris P.A., Taylor R., Minor B.L., Elliott V., Fernandez M., O’Neal L., McLeod L., Delacqua G., Delacqua F., Kirby J. (2019). The REDCap Consortium: Building an International Community of Software Platform Partners. J. Biomed. Inform..

[16] Blog 51-Chronology of the Corona Crisis in Austria-Part 1: Background, the Way to the Lockdown, the Acute Phase and Economic Consequences. https://viecer.univie.ac.at/en/projects-and-cooperations/austrian-corona-panel-project/corona-blog/corona-blog-beitraege/blog51/.

[17] Probst T., Humer E., Stippl P., Pieh C. (2020). Being a Psychotherapist in Times of the Novel Coronavirus Disease: Stress-Level, Job Anxiety, and Fear of Coronavirus Disease Infection in More Than 1,500 Psychotherapists in Austria. Front. Psychol..

[18] Blog 112 (EN)-Chronology of the Corona Crisis in Austria-Part 5: Third Wave, Regional Lockdowns and the Vaccination Campaign. https://viecer.univie.ac.at/en/projects-and-cooperations/austrian-corona-panel-project/corona-blog/corona-blog-beitraege/blog112-en/.

[19] Nussbaumer-Streit B., Jesser A., Humer E., Barke A., Doering B.K., Haid B., Schimböck W., Reisinger A., Gasser M., Eichberger-Heckmann H. (2022). A Web-Survey Assessed Attitudes toward Evidence-Based Practice among Psychotherapists in Austria. Sci. Rep..

[20] Probst T., Humer E., Jesser A., Pieh C. (2022). Attitudes of Psychotherapists towards Their Own Performance and the Role of the Social Comparison Group: The Self-Assessment Bias in Psychodynamic, Humanistic, Systemic, and Behavioral Therapists. Front. Psychol..

[21] Blog 150-Chronologie zur Corona-Krise in Österreich-Teil 7: Der Delta-Lockdown, die Omikron-Welle und das “Frühlingserwachen”. https://viecer.univie.ac.at/corona-blog/corona-blog-beitraege/blog-150-chronologie-zur-corona-krise-in-oesterreich-teil-7-der-delta-lockdown-die-omikron-welle-und-das-fruehlingserwachen/.

[22] Schaffler Y., Kaltschik S., Probst T., Jesser A., Pieh C., Humer E. (2022). Mental Health in Austrian Psychotherapists during the COVID-19 Pandemic. Front. Public Health.

[23] Heidegger K.-E. The Situation of Psychotherapy in Austria. http://www.europsyche.org/situation-of-psychotherapy-in-various-countries/austria/.

[24] Crowe M., Inder M., Porter R. (2015). Conducting Qualitative Research in Mental Health: Thematic and Content Analyses. Aust. N. Z. J. Psychiatry.

[25] (2022). ATLAS.Ti 22 Windows.

[26] Dale R., Budimir S., Probst T., Stippl P., Pieh C. (2021). Mental Health during the COVID-19 Lockdown over the Christmas Period in Austria and the Effects of Sociodemographic and Lifestyle Factors. Int. J. Environ. Res. Public Health.

[27] Humer E., Schaffler Y., Jesser A., Probst T., Pieh C. (2022). Mental Health in the Austrian General Population during COVID-19: Cross-Sectional Study on the Association with Sociodemographic Factors. Front. Psychiatry.

[28] Singh S., Sagar R. (2022). Online Psychotherapy During the COVID-19 Pandemic: The Good, the Bad, and the Ugly. Indian J. Psychol. Med..

[29] Andrews G., Basu A., Cuijpers P., Craske M.G., McEvoy P., English C.L., Newby J.M. (2018). Computer Therapy for the Anxiety and Depression Disorders Is Effective, Acceptable and Practical Health Care: An Updated Meta-Analysis. J. Anxiety Disord..

[30] Luo C., Sanger N., Singhal N., Pattrick K., Shams I., Shahid H., Hoang P., Schmidt J., Lee J., Haber S. (2020). A Comparison of Electronically-Delivered and Face to Face Cognitive Behavioural Therapies in Depressive Disorders: A Systematic Review and Meta-Analysis. eClinicalMedicine.

[31] Jesser A., Muckenhuber J., Lunglmayr B., Dale R., Humer E. (2021). Provision of Psychodynamic Psychotherapy in Austria during the COVID-19 Pandemic: A Cross-Sectional Study. Int. J. Environ. Res. Public Health.

[32] Simpson S. (2009). Psychotherapy via Videoconferencing: A Review. Br. J. Guid. Couns..

[33] Simpson S., Bell L., Knox J., Mitchell D. (2005). Therapy via Videoconferencing: A Route to Client Empowerment?. Clin. Psychol. Psychother..

[34] Simpson S., Knox J., Mitchell D., Ferguson J., Brebner J., Brebner E. (2003). A Multidisciplinary Approach to the Treatment of Eating Disorders via Videoconferencing in North-East Scotland. J. Telemed. Telecare.

[35] Coroiu A., Moran C., Campbell T., Geller A.C. (2020). Barriers and Facilitators of Adherence to Social Distancing Recommendations during COVID-19 among a Large International Sample of Adults. PLoS ONE.

[36] Lin T., Harris E.A., Heemskerk A., Van Bavel J.J., Ebner N.C. (2021). A Multi-National Test on Self-Reported Compliance with COVID-19 Public Health Measures: The Role of Individual Age and Gender Demographics and Countries’ Developmental Status. Soc. Sci. Med..

[37] Schaffler Y., Probst T., Jesser A., Humer E., Pieh C., Stippl P., Haid B., Schigl B. (2022). Perceived Barriers and Facilitators to Psychotherapy Utilisation and How They Relate to Patient’s Psychotherapeutic Goals. Healthcare.

[38] Schigl B., Lerch L., Rohner J. (2021). Erfahrungen von Wiener Psychotherapeut_innen mit der Antragstellung und Bewilligungspraxis der Krankenkassen. Psychother. Forum.

